# Supplementary figures and images for: Neuropeptidergic transmission shapes emergent properties of prefrontal cortical circuits underlying learning
Source: bioRxiv. 2025 May 13:2025.05.13.653840. Preprint. [Version 1] doi: 10.1101/2025.05.13.653840 (PMC12132504; doi:10.1101/2025.05.13.653840)

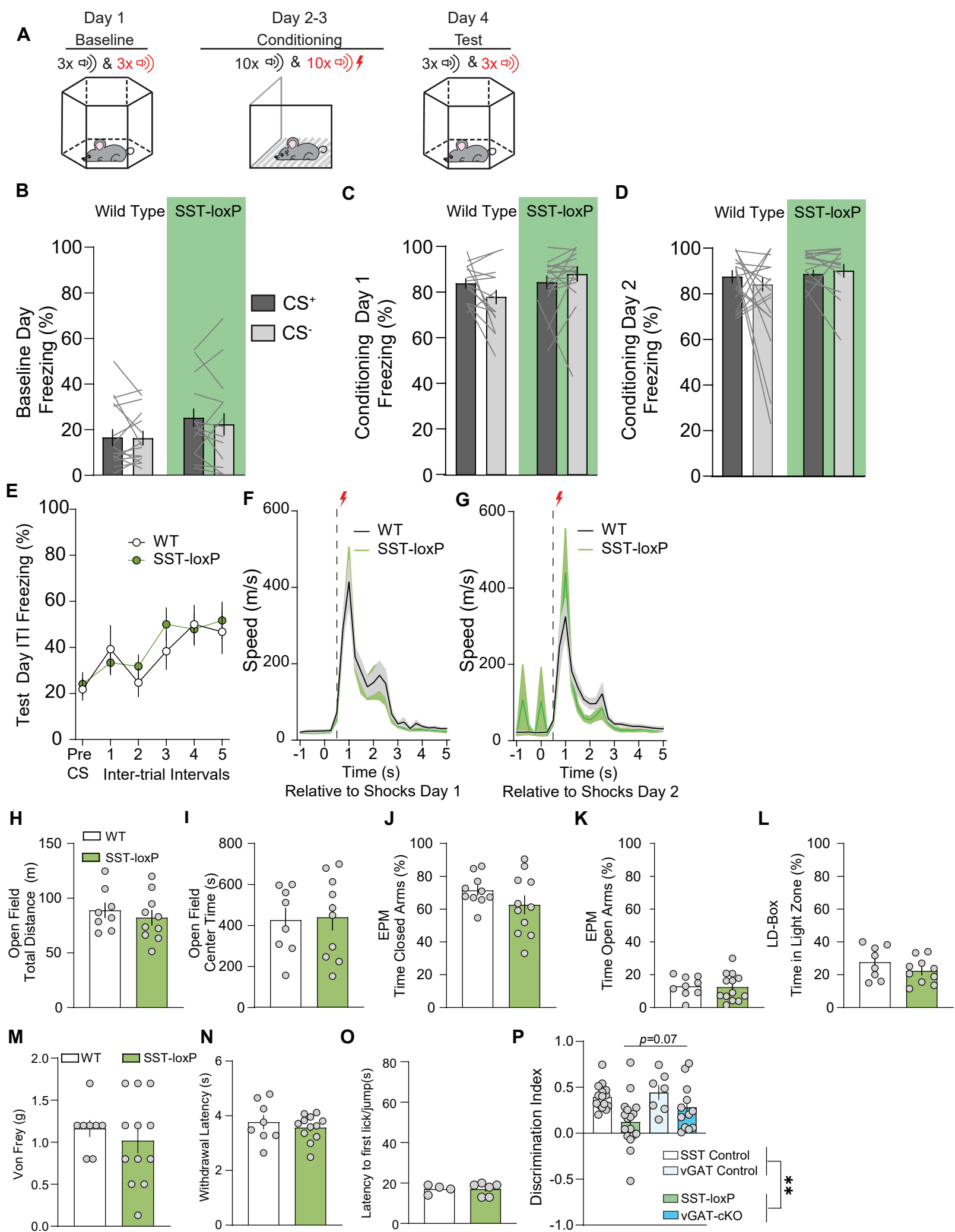

Supplement: Supplement 1 — Figure S1: Related to figure 1: mPFCSST knockdown impairs cued auditory threat discrimination. (A) Experimental timeline for cued threat discrimination task. (B) Freezing during the baseline session in control and mPFCSST-cKO mice (Two-way ANOVA, Genotype x Trial Interaction, p=0.8222). (C,D) Freezing during conditioning day 1 (C; Two-way ANOVA, Genotype x Trial Interaction, p=0.0988) and conditioning day 2 (D; Two-way ANOVA, Genotype x Trial Interaction, p=0.8403) of cued threat discrimination in control and mPFCSST-cKO mice. (E) Test day freezing during the ITI in control and mPFCSST-cKO mice (Two-way ANOVA, Genotype x Trial Interaction, p=0.7973). (F,G) Similar changes in speed evoked by footshocks during conditioning day 1 (E; Two-way ANOVA, Genotype x Trial Interaction, p>0.0001) and conditioning day 2 (G; Two-way ANOVA, Genotype x Trial Interaction, p=0.5047) of threat discrimination in control and SST-loxP mice. (H,I) Open field total distance traveled (H; Unpaired t-test p=0.5034) and center time (I; Unpaired t-test p=0.8764). (J,K) Elevated plus maze time in closed arms (J; Unpaired t-test p=0.2059) and open arms (K; Unpaired t-test p=0.8695). (L) Percent time spent in the light zone in the light-dark box assay (Unpaired t-test p=0.2266). (M) von Frey filament threshold in grams (g; Unpaired t-test p=0.5318). (N) Cold plate withdrawal latency in seconds (s; Unpaired t-test p=0.6222). (O) Hotplate lick/jump latency in seconds (s; Unpaired t-test p=0.8107). (P) Discrimination index (CS−/CS+ test day freezing) in SST control (white), mPFCSST-cKO (green), vGAT-cKO control (light blue), and vGAT-cKO (blue) during test day (Two-way ANOVA, Manipulation x Transmitter Interaction, p=0.4267, Manipulation Main Effect, **p=0.0027; Tukey’s Post Hoc test, p=0.0746 SST-loxP vs vGAT-cKO). [file media-1.pdf]

**A**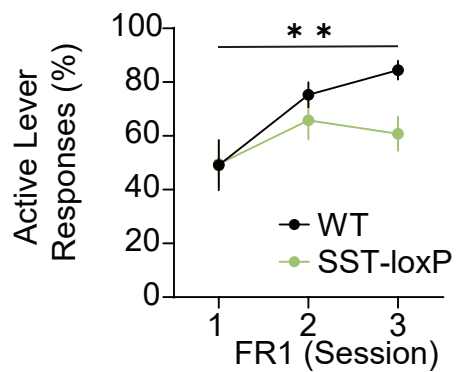**B**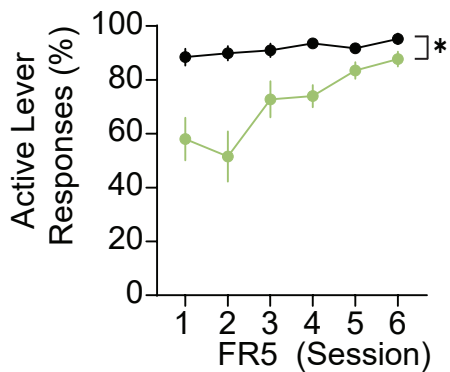**C**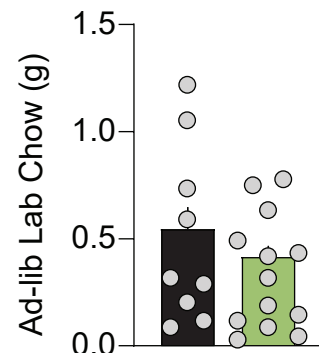**D**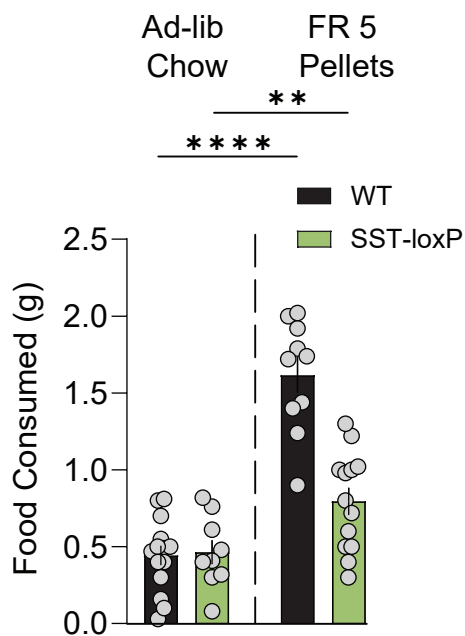**E**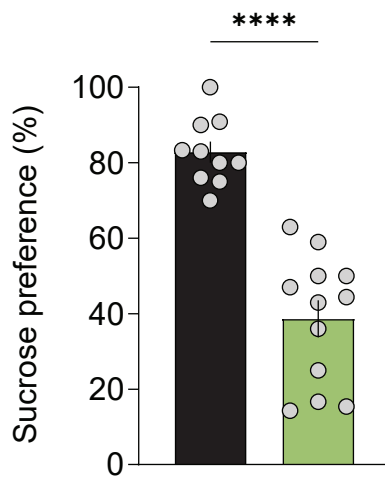

Supplement: Supplement 2 — Figure S2: related to figure 2 mPFC SST peptidergic regulation of discrimination learning of novel and appetitive outcomes. (A,B) Percentage of active lever (active lever presses / (active + inactive lever presses)) during FR1 (A; Two-way ANOVA, Days Main Effect *p=0.0036, Genotype Main Effect *p=0.0148) and FR5 (Two-way ANOVA with Tukey’s Post Hoc test, *p=0.0168 WT lever accuracy vs mPFCSST-cKO lever accuracy day 1; *p=0.0300 WT lever accuracy vs mPFCSST-cKO lever accuracy day 4) sessions in control and mPFCSST-cKO mice. (C) Freely available chocolate pellets consumed during exposure to chocolate pellets in the operant chamber without the active and inactive levers (Unpaired t-test p=0.4801). D) Total freely available laboratory chow and operant-derived chocolate pellets in grams (g; One-way ANOVA with Tukey’s Post Hoc test, ****p<0.0001 WT Ad-libitum vs WT FR5, **p=0.0165 mPFCSSTAd-libitum vs mPFCSST FR5). E) Sucrose preference in WT and SST-loxP mice (Unpaired t-test, ****p<0.0001). [file media-2.pdf]

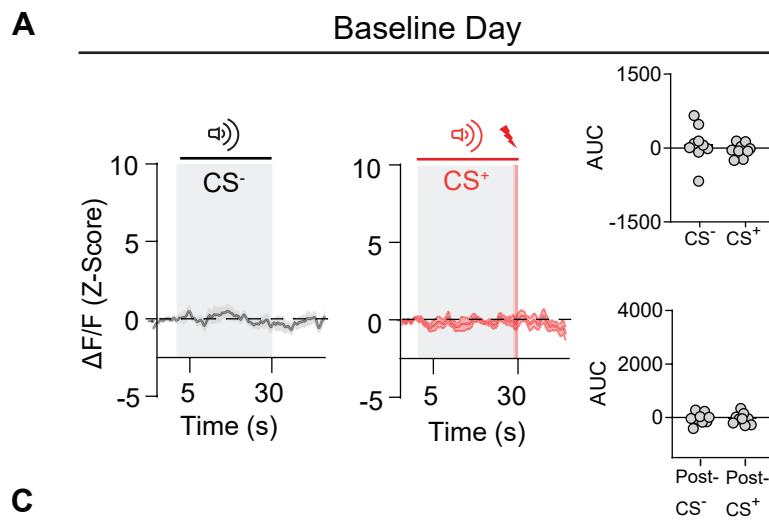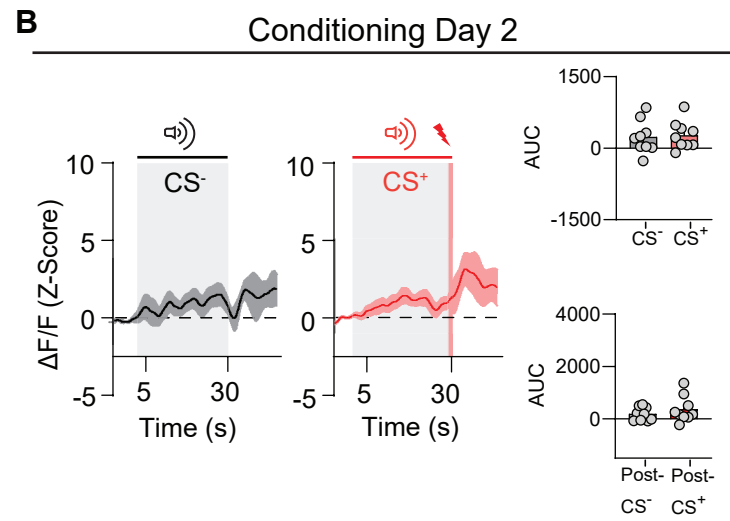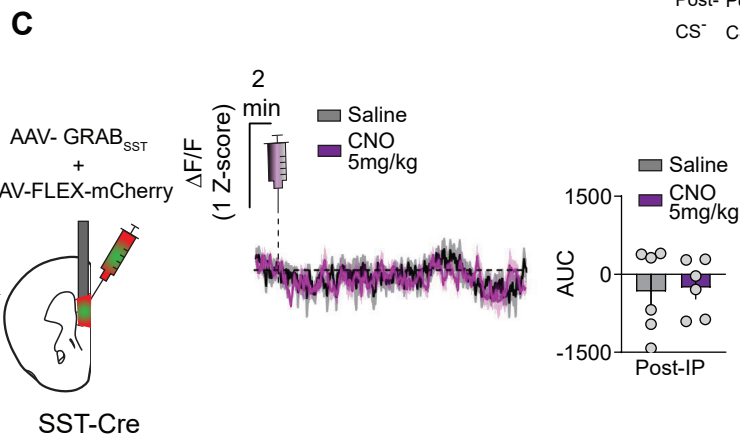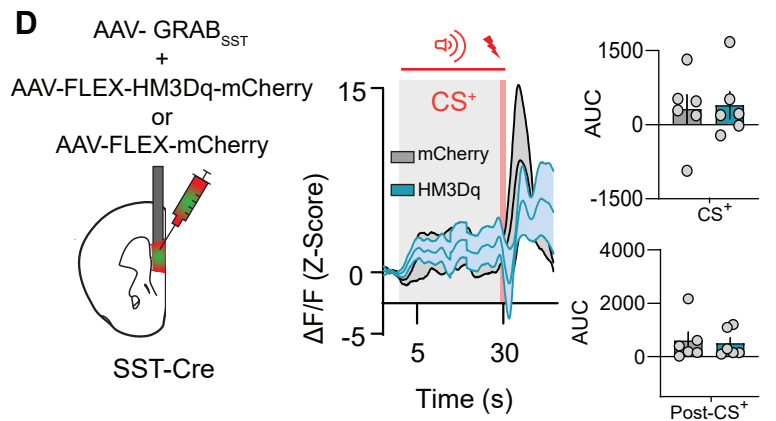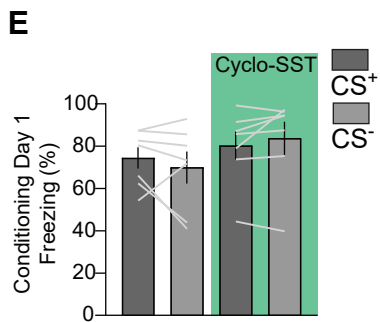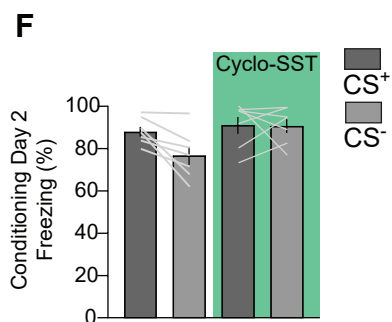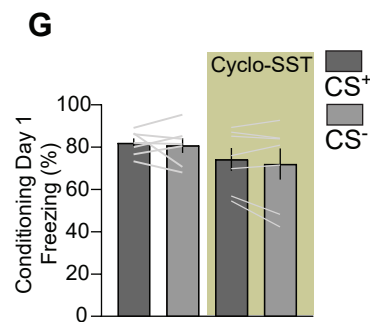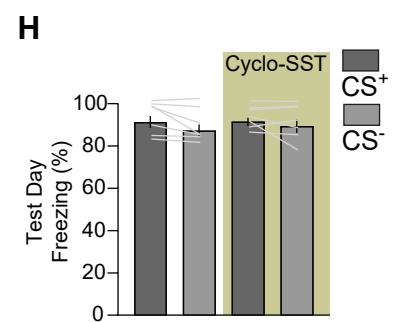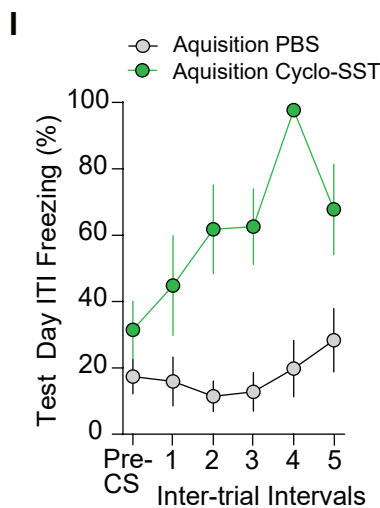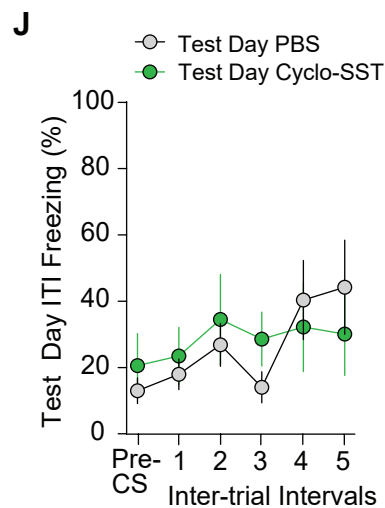

Supplement: Supplement 4 — Figure S4: Related to Figure 4. mPFC SST release during cued auditory threat discrimination promotes discriminative learning A) GRABSST-mediated fluorescence during presentation of the CS− (left) and CS+ (right) during baseline day. AUC of the time windows for the CS+ and CS− (top; Paired t-test p=0.4004) and the post-CS period (bottom; Paired t-test p=0.8853). B) Same as in A but during conditioning day 2. AUC of the time windows for the CS+ and CS− (top; Paired t-test p=0.7987) and post-CS periods (bottom; Paired t-test p=0.3343). C) Time course of GRABSST fluorescence in response to CNO and saline treatment in mice expressing AAV-GRAB-SST and AAV-FLEX-mCherry (Paired t-test p=0.8510). D) Footshock-evoked responses in mCherry-expressing control mice and HM3Dq expressing to demonstrate control mice had functional GRABSST expression. AUC of the time windows for the CS+ and CS− (top; Paired t-test p=0.8422) and post-CS periods (bottom; Paired t-test p=0.8146). E-H) Freezing during CS+ and CS− during threat discrimination conditioning day 1 (E; Two-way ANOVA, Genotype x Trial Interaction, p=0.2138) and conditioning day 2 (F; Two-way ANOVA, Genotype x Trial Interaction, p=0.0882) in mice injected with Cyclo-SST during conditioning day 1. Freezing during CS+ and CS− during threat discrimination conditioning day 1 (G; Two-way ANOVA, Genotype x Trial Interaction, p=0.8125) and conditioning day 2 (H; Two-way ANOVA, Genotype x Trial Interaction, p=0.5053) in mice injected with Cyclo-SST during test day. I) Test day freezing during the ITI in mice treated with Cyclo-SST on conditioning day 1 (Two-way ANOVA, Genotype x Trial Interaction, *p=0.02237). J) Test day freezing during the ITI in mice treated with Cyclo-SST on test recall day (Two-way ANOVA, Genotype x Trial Interaction, p=0.5360). [file media-4.pdf]

## Test Day CS<sup>+</sup> Modulated

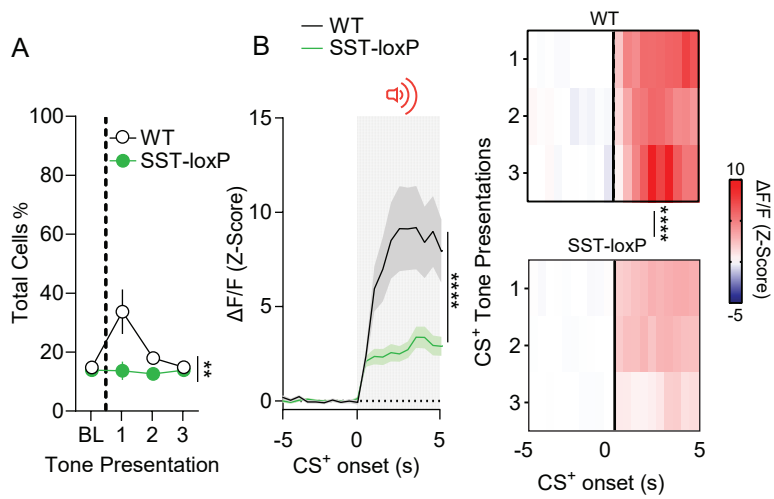

## Test Day CS<sup>-</sup> Modulated

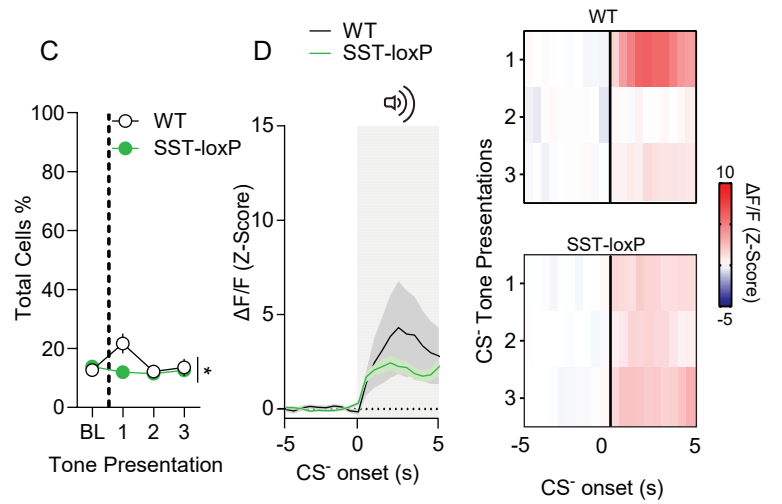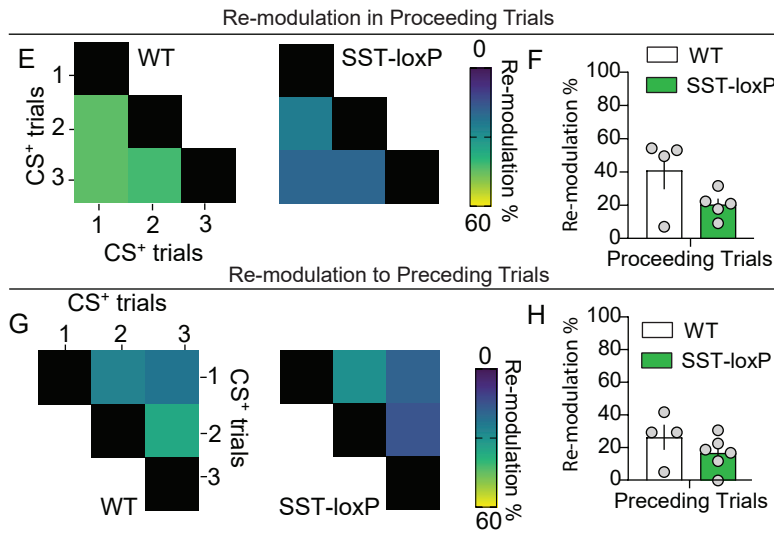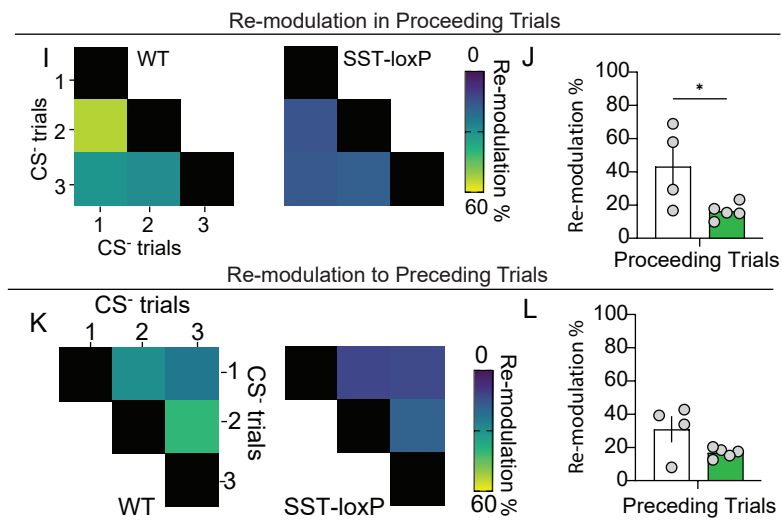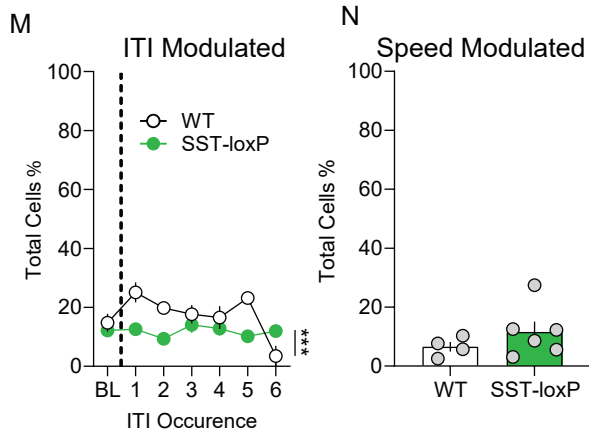

Supplement: Supplement 7 — Figure S7: Related figure 5: mPFC SST knockdown alters mPFC neuronal encoding in a cued-threat discrimination task (A, C, M, N) Percentage of total neurons modulated by CS+ (A), CS− (C), ITI (M), and speed (N) during test day in WT and SST-loxP mice (A, Two-way ANOVA, Tone x Genotype Interaction **p=0.0050; C, Two-way ANOVA, Tone x Genotype Interaction *p=0.0278; M, Two-way ANOVA Tone x Genotype Interaction ***p=0.0003; N, Unpaired t-test, p=0.3019). (B,D) Timecourse of Z-scored GCaMP7f activity of neurons modulated by CS+ (B) and CS− (D) during test day in WT and SST-loxP mice. Heatmaps representing Z-scored activity in response to CS+ and CS− across trials in WT (top) and SST-loxP (bottom) mice. (B; Two-way ANOVA, Time x Genotype Interaction ****p<0.0001; D, Two-way ANOVA, Time x Genotype Interaction p=0.2210). (E,F,I,J) Heatmap representing the percent of neurons modulated by CS+ (E) or CS− (I) tones across test day in WT (left) and SST-loxP (right) neurons. Re-modulation percentage was calculated by determining the percentage of neurons with significant modulation in a specific trial (CS+n or CS−n) that were also significantly modulated in n-proceeding (CSn+i) trials. % Re-modulation = (CSn | CSn±i / CSn), (F, Unpaired t-test p=0.0975; J, Unpaired t-test *p=0.0436). (G,H, K, L) Heatmap representing the percent of neurons modulated by CS+ (G) or CS− (K) tones across test day in WT (left) and SST-loxP (right) neurons. Remodulation percentage was calculated by determining the percentage of neurons with significant modulation in a specific trial (CS+n or CS−n) that were also significantly modulated n-preceding (CSn-i) trials (CSn | CSn±i). % re-modulation = (CSn | CSn±i / CSn), (H, Unpaired t-test p=0.2654; L, Unpaired t-test p=0.0856). [file media-7.pdf]

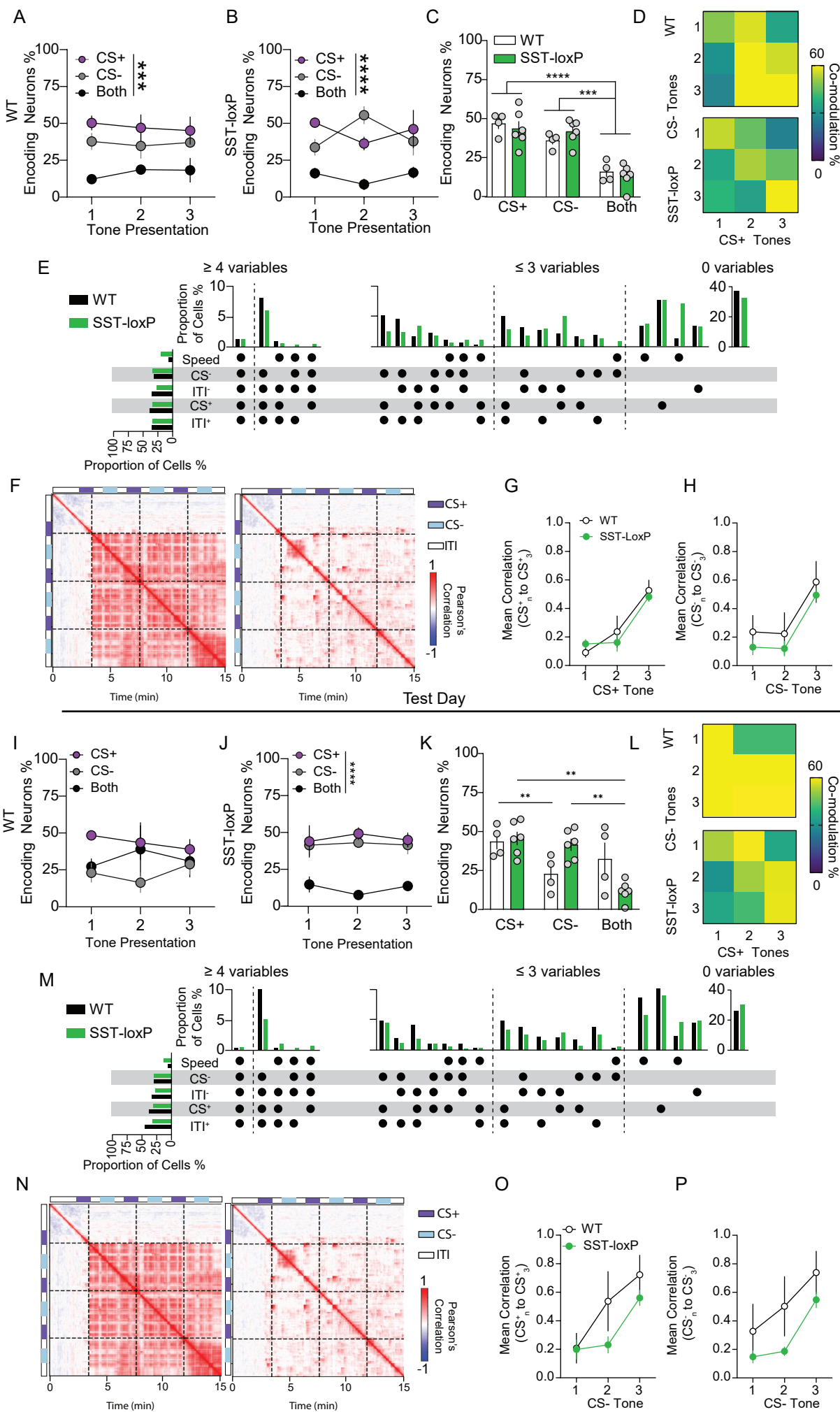

Supplement: Supplement 8 — Figure S8: Related to Figure 6: mPFC SST knockdown alters encoding of task variables in a threat discrimination task. (A,B) Baseline day data representing the percentage of tones encoding the CS+, CS−, or both tones across blocks of CS+/CS− pairs in WT mice (A, Two-way ANOVA, Encoding Type Main Effect, ***p=0.0002) and SST-loxP mice (B, Two-way ANOVA, Encoding Type Main Effect, ****p<0.0001). (C) Average CS+, CS−, and encoding of both CS collapsed across presentations in WT and SST-loxP mice (Two-way ANOVA, Encoding Type Main Effect, ****p<0.0001; Tukey’s Main Effects Comparison; CS+ vs both, ****p<0.0001, CS− vs both, ***p=0.0002). (D) Heatmap representing the percent of neurons activated during both the CS+ and CS− tones across the baseline day session in WT (top) and SST-loxP (bottom) neurons. Co-modulation percentage is calculated as the number of neurons with significant modulation to CS+ in a given trial and all CS− trials. (E) UpSet visualization of neurons modulated by different combinations of footshock, CS−, CS+ or ITI following a CS+ (ITI+) and CS− (ITI−). Categories with <1% of neurons modulated are not shown. Left horizontal bars represent percentages of cardinal categories, including those not shown. Dashed lines indicate separation of categories encoding different numbers of variables. (F) Heatmaps representing Pearson’s correlation of neural activity across the baseline day session for WT and SST-loxP mice. Red values indicate higher correlation, and blue values indicate lower correlation. (G) Pearson’s correlation of activity from CS+ tones 1-3 to the last tone, as represented in F (Two-way ANOVA, Genotype Presentation Main Effect p=0.7654). (H) Pearson’s correlation of activity from CS− tones 1-3 to the last tone, as represented in F (Two-way ANOVA, Genotype Presentation Main Effect p=0.3668). (I,J) Test day data representing the percentage of tones encoding the CS+, CS−, or both tones across blocks of CS+/CS− pairs in WT mice (I, Two-way ANOVA, En [file media-8.pdf]

## Conditioning Day 2

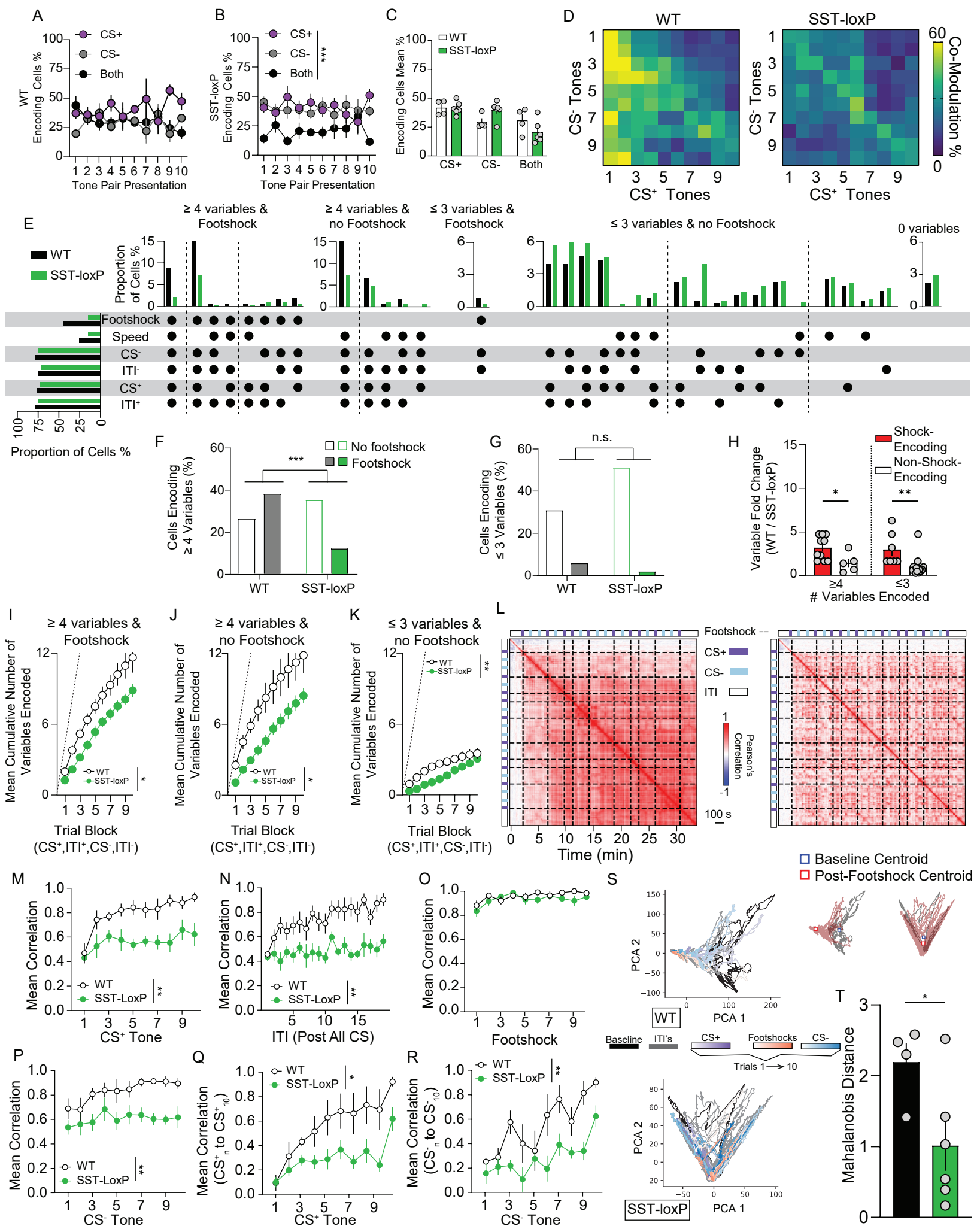

Supplement: Supplement 9 — Figure S9: Related to Figure 6: mPFC SST knockdown alters encoding of task variables in a threat discrimination task. (A,B) Conditioning day 2 data representing the percentage of tones encoding the CS+, CS−, or both tones across blocks of CS+/CS− pairs in WT mice (A, Two-way ANOVA, Encoding Type Main Effect, p=0.1252) and SST-loxP mice (B, Two-way ANOVA, Encoding Type Main Effect, ***p=0.0005). (C) Average CS+, CS−, and encoding of both CS collapsed across presentations in WT and SST-loxP mice (Two-way ANOVA, Encoding Type Main Effect, *p=0.0220). (D) Percentage of neurons modulated by the CS+ and CS− tone across the conditioning day 2 session in WT (left) and SST-loxP (right) mice. Co-modulation percentage is calculated as the number of neurons with significant modulation to CS+ in a given trial and all CS− trials. (E) UpSet visualization of neurons modulated by different combinations of footshock, CS−, CS+ or ITI following a CS+ (ITI+) and CS− (ITI−). Categories with <1% of neurons modulated are not shown. Left horizontal bars represent percentages of cardinal categories, including those not shown. Dashed lines indicate separation of categories encoding different numbers of variables. (F, G) Percentage of neurons with configural encoding (≥4 variables; F, left; Two-sided Fishers Exact Test ***p=0.0005) or with limited encoding (≤3 variables; G, right; Two-sided Fishers Exact Test p=0.0603) including and excluding footshock in WT and SST-loxP mice. (H) The variable fold difference between WT and SST-loxP mice in distinct categories (each represented by a dot) containing (red) or lacking a footshock (gray) with configural encoding (≥4 variables; left) or limited encoding (≤3 variables; right) of variables. Fold difference is the percentage of neurons encoding a distinct category in WT mice divided by the percentage of neurons in SST-loxP mice. Fold differences greater and less than 1 indicate more encoding in WT and SST-loxP mice for a category, respectively (Two-wa [file media-9.pdf]
