# Supplementary material for: Neuropeptidergic transmission shapes emergent properties of prefrontal cortical circuits underlying learning
Source: bioRxiv. 2025 May 13:2025.05.13.653840. Preprint. [Version 1] doi: 10.1101/2025.05.13.653840 (PMC12132504; doi:10.1101/2025.05.13.653840)
Supplement: Supplement 3 — Fig. S3: Related to Figure 3. mPFCSST interneurons encode footshock outcomes, neutral and threat-predictive cues, and post-threat periods. (A,K) CS modulation of mPFCSST neuron activity across CS+ and CS− tone presentations during the baseline session (A; Two-way ANOVA, Time Main Effect, *p=0.0136) and conditioning day 2 (K; Two-way ANOVA, Time Main Effect, ****p <0.0001). (B, O) Z-Scored Ca2+ activity in CS+ and CS− modulated neurons across all trials during the baseline session (B; Two-way ANOVA, Time x Stimulus Interaction *p<0.0212) and conditioning day 2 (O; Two-way ANOVA, Time x Stimulus Interaction ****p<0.0001). (C,M) Percentage of neurons whose activity is modulated during CSn and also subsequently modulated in proceeding trials CSn+I during baseline (C) and conditioning day 2 (M). Mean percentage of neurons modulated in subsequent trials in baseline day (C; Paired t-test, p=0.4529) and conditioning day 2 (M; p=0.9015). (D,N) Same as C but for neurons whose activity was modulated during CSn and during preceding trials CSn-I. Mean percentage of neurons modulated in subsequent trials in baseline day (D; Paired t-test, p=0.1369) and conditioning day 2 (N; p=0.3409). (E, Q) Percentage of neurons whose activity was modulated during the ITI following a CS+ (ITI+) and CS− (ITI−) during the baseline session (Two-way ANOVA, time main effect *p=0.0484) and conditioning day 2 (Two Way ANOVA, time main effect ****p<0.0001). (F, R) Ca2+ activity in ITI+ and ITI− modulated neurons collapsed across trials during baseline session (F; Two-way ANOVA, Time x ITI Type Interaction, p=0.8835) and conditioning session day 2 (R; Two-way ANOVA, Time x ITI Type Interaction, ****p<0.0001). (G,S) Percentage of neurons modulated by speed during the baseline session (G) and conditioning day 2 (S). (H,T) Speed modulated neurons did not respond to changes in speed in a linear manner during the baseline session (H) and conditioning day 2 (T; See statistics table for Pearson Correlation coe [file media-3.pdf]

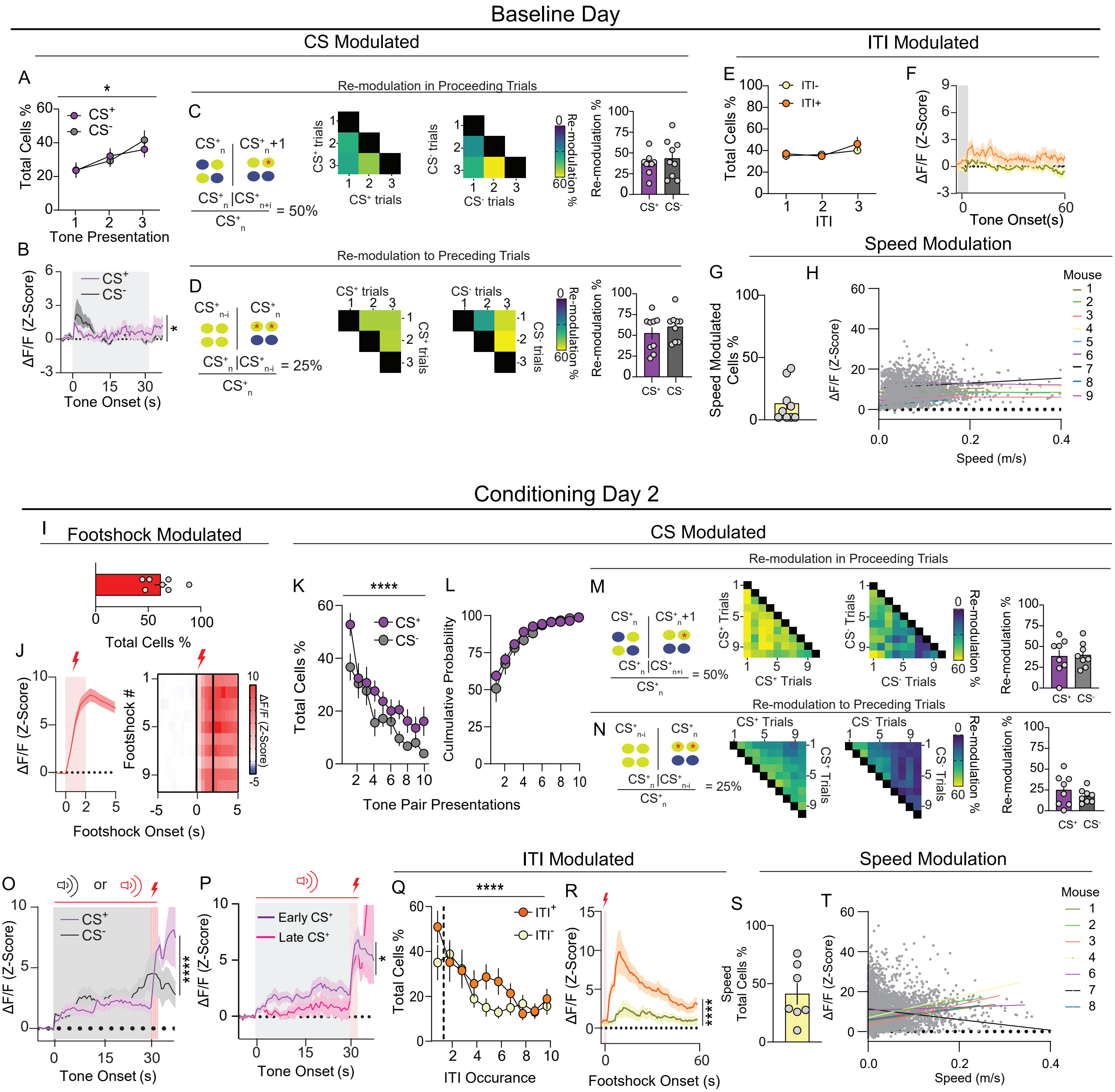
Baseline Day
CS Modulated
ITI Modulated
A
Total Cells %
CS+
CS-
Tone Presentation
B
ΔF/F (Z-Score)
Tone Onset (s)
C
Re-modulation in Proceeding Trials
CS+n |CS+n+i / CS+n = 50%
CS+ trials
CS- trials
Re-modulation %
D
Re-modulation to Preceding Trials
CS+n |CS+n-i / CS+n = 25%
E
ITI-
ITI+
ITI
F
Tone Onset(s)
Speed Modulation
G
Speed Modulated Cells %
H
Mouse
Speed (m/s)
Conditioning Day 2
I
Footshock Modulated
Total Cells %
J
Footshock #
Footshock Onset (s)
K
L
Culmulative Probability
Tone Pair Presentations
M
CS+ Trials
CS- Trials
N
O
or
P
Early CS+
Late CS+
Q
ITI Occurance
R
Footshock Onset (s)
S
Speed Total Cells %
T
