# Supplementary material for: Neuropeptidergic transmission shapes emergent properties of prefrontal cortical circuits underlying learning
Source: bioRxiv. 2025 May 13:2025.05.13.653840. Preprint. [Version 1] doi: 10.1101/2025.05.13.653840 (PMC12132504; doi:10.1101/2025.05.13.653840)
Supplement: Supplement 5 — Figure S5: Related figure 5: mPFC SST knockdown alters mPFC neuronal encoding in a cued-threat discrimination task (A,C,M,N) Percentage of total neurons modulated by CS+ (A), CS− (C), ITI (M), and speed (N) during baseline day, WT vs SST-loxP (A, Two-way ANOVA, Time x Genotype Interaction p=0.1422; C, Two-way ANOVA, Time x Genotype interaction p=0.6835; M, Two-way ANOVA Time x Genotype Interaction p=0.8999; N, Unpaired t-test, p=0.1694). (B,D) Timecourse of Z-scored GCaMP7f activity of neurons modulated by CS+ (B), and CS− (D), during baseline day in WT and SST-loxP. Heatmaps representing Z-scored activity in CS+ and CS− across trials in WT (top) and SST-loxP (bottom) mice. (B; Two-way ANOVA, time x genotype interaction p= 0.1486, D; Two-way ANOVA, Time x Genotype Interaction p=0.0691). (E,F,I,J) Heatmap representing the percent of neurons modulated by CS+ (A) or CS− (C) tones across baseline day in WT (left) and SST-loxP (right) mice. Re-modulation percentage was calculated by determining the percentage of neurons with significant modulation in a specific trial (CS+n or CS−n) that were also significantly modulated in n-proceeding (CSn+i) trials. % Re-modulation = (CSn | CSn±i / CSn), (F, Unpaired t-test p=0.3808; J, Unpaired t-test p=0.1353). (G,H, K, L) Heatmap representing the percent of neurons modulated by CS+ (I) or CS− tones across baseline day in WT (left) and SST-loxP (right) mice. Re-modulation percentage was calculated by determining the percentage of neurons with significant modulation in a specific trial (CS+n or CS−n) that were also significantly modulated n-preceding (CSn-i) trial (CSn | CSn±i) trials. % Re-modulation = (CSn | CSn±i / CSn), (H, Unpaired t-test p=0.5974; L, Unpaired t-test p=0.2882). [file media-5.pdf]

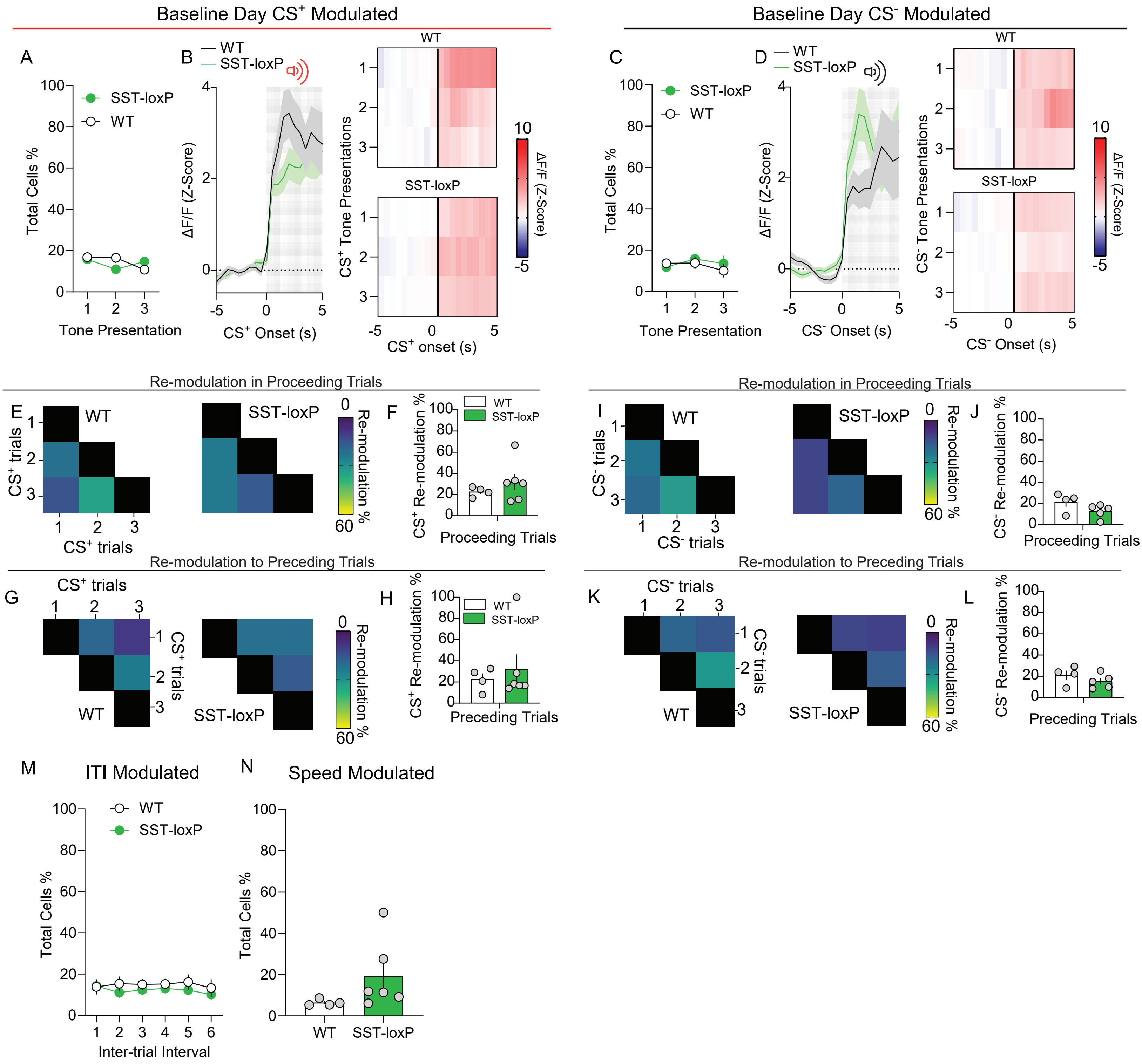

Baseline Day CS+ Modulated
Baseline Day CS- Modulated
A
B
C
D
SST-loxP
WT
Total Cells %
Tone Presentation
ΔF/F (Z-Score)
CS+ Onset (s)
CS+ Tone Presentations
CS+ onset (s)
CS- Onset (s)
CS- Tone Presentations
ΔF/F (Z-Score)
Re-modulation in Proceeding Trials
Re-modulation to Preceding Trials
E
F
G
H
I
J
K
L
CS+ trials
CS- trials
Re-modulation %
CS+ Re-modulation %
CS- Re-modulation %
Proceeding Trials
Preceding Trials
M
ITI Modulated
N
Speed Modulated
Inter-trial Interval
