# Supplementary material for: Neuropeptidergic transmission shapes emergent properties of prefrontal cortical circuits underlying learning
Source: bioRxiv. 2025 May 13:2025.05.13.653840. Preprint. [Version 1] doi: 10.1101/2025.05.13.653840 (PMC12132504; doi:10.1101/2025.05.13.653840)
Supplement: Supplement 6 — Figure S6: Related figure 5: mPFC SST knockdown alters mPFC neuronal encoding in a cued-threat discrimination task (A,C,O,Q,R) Percentage of total neurons modulated by CS+ (A), CS− (C), footshock (O), ITI (Q), and speed (R) during conditioning day 2 in WT and SST-loxP mice (A, Two-way ANOVA, Time x Genotype Interaction ****p<0.0001; C, Two-way ANOVA, Time x Genotype Interaction ***p=0.0003; O, Unpaired t-test, **p=0.0031; Q, Two-way ANOVA Time x Genotype Interaction ****p<0.0001; R, Unpaired t-test, p=0.6603). (B,D,P) Timecourse of Z-scored GCaMP7f activity of neurons modulated by CS+ (B), and CS− (D), and footshock (P) during conditioning day 2 in WT and SST-loxP mice. Heatmaps representing Z-scored activity in response to CS+ and CS− across trials in WT (top) and SST-loxP (bottom) mice. (B, Two-way ANOVA, Time x Genotype Interaction ****p<0.0001; D, Two-way ANOVA, Time x Genotype Interaction ***p=0.0008; P, Two-way ANOVA, Time x Genotype Interaction ***p=0.0003). (E, H) Cumulative probability of cells that become significantly modulated by the CS+ (E) and CS− (H) across discrimination day 2 (E; Two-way ANOVA, Genotype Main Effect ***p=0.001; H, Two-way ANOVA, Genotype Main Effect **p=0.0042). (F,G, I, H) Heatmap representing the percent of neurons modulated by CS+(F,G) or CS−(I,J) tones across conditioning day 1 session in WT (left) and SST-loxP (right) neurons. Re-modulation percentage was calculated by determining the percentage of neurons with significant modulation in a specific trial (CS+n or CS−n) that were also significantly modulated in n-proceeding (CSn+i) or n-preceding (CSn-i) trials (CSn | CSn±i). % Re-modulation = (CSn | CSn±i / CSn) (K, L) Percentage of neurons with significant modulation in n-proceeding trials WT vs SST-loxP (K, Unpaired t-test **p=0.0065; L, Unpaired t-test *p=0.0191). (M, N) Percentage of neurons with significant modulation in n-preceding trials WT vs SST-loxP (M, Unpaired t-test p=0.2296; N, Unpaired t-test p=0.7899). [file media-6.pdf]

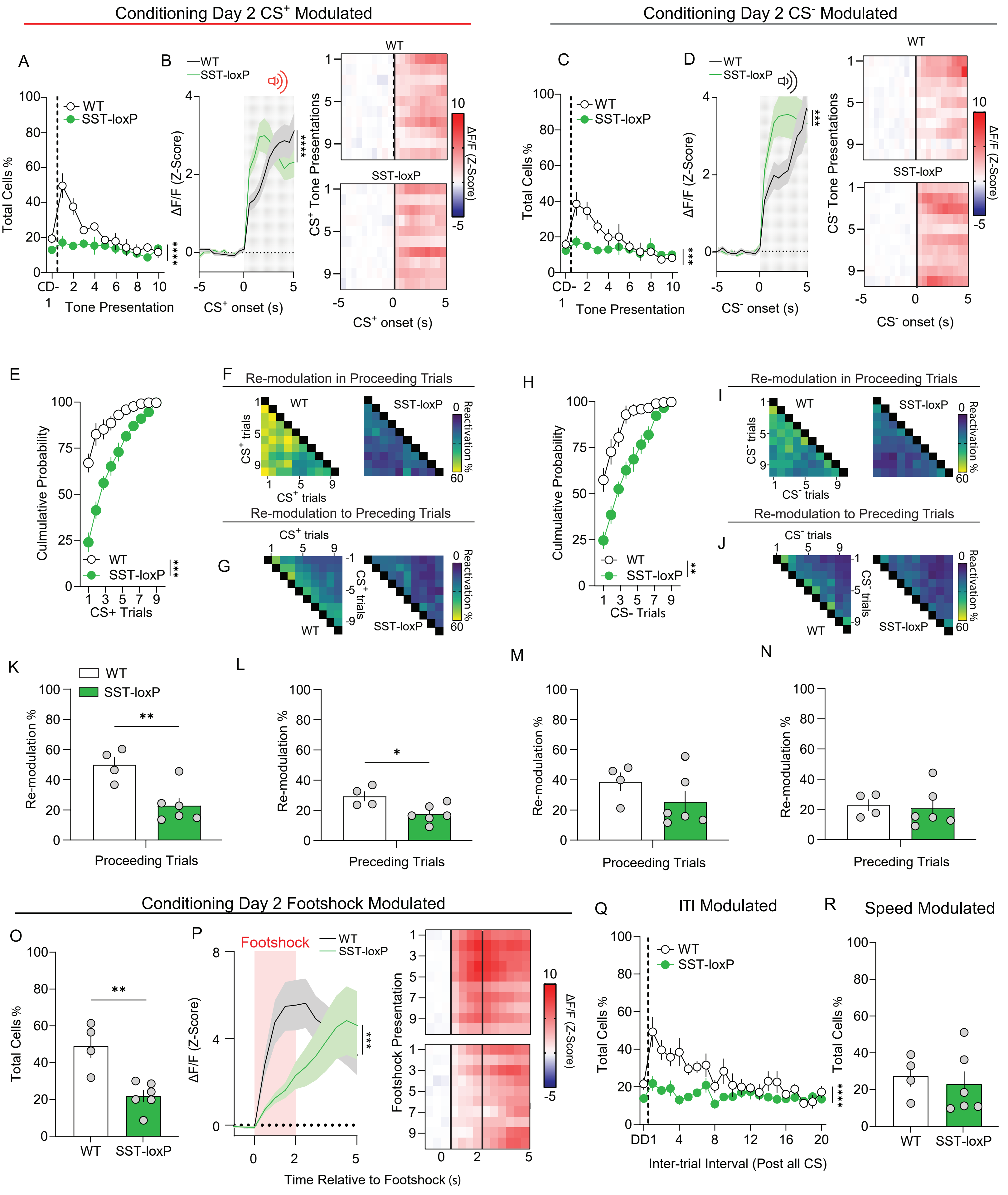
Conditioning Day 2 CS+ Modulated
Conditioning Day 2 CS- Modulated
A
B
C
D
Total Cells %
Tone Presentation
WT
SST-loxP
ΔF/F (Z-Score)
CS+ onset (s)
CS+ Tone Presentations
CS- onset (s)
CS- Tone Presentations
E
F
G
H
I
J
Re-modulation in Proceeding Trials
Re-modulation to Preceding Trials
Reactivation %
CS+ trials
CS- trials
Culmulative Probability
CS+ Trials
CS- Trials
K
L
M
N
Re-modulation %
Proceeding Trials
Preceding Trials
Conditioning Day 2 Footshock Modulated
O
P
Q
R
Footshock
Time Relative to Footshock (s)
Footshock Presentation
ITI Modulated
Inter-trial Interval (Post all CS)
Speed Modulated
