# Supplementary material for: Neuropeptidergic transmission shapes emergent properties of prefrontal cortical circuits underlying learning
Source: bioRxiv. 2025 May 13:2025.05.13.653840. Preprint. [Version 1] doi: 10.1101/2025.05.13.653840 (PMC12132504; doi:10.1101/2025.05.13.653840)
Supplement: Supplement 10 — Figure S10: Related to Figure 6: mPFC SST knockdown alters encoding of task variables in a threat discrimination task. (A-D) Mean CS+/ CS− co-modulation values across all trials for encoding cells represented in co-modulation heatmap for proceeding and preceding trials during baseline (A; Fig. S8D; Two-way ANOVA, Proceeding vs Preceding x Genotype Interaction, p=0.8868), conditioning day 1 (B; Fig. 6D; Two-way ANOVA, Proceeding vs Preceding x Genotype Interaction, *p=0.0180; Tukey’s Post-hoc test WT vs SST-loxP Proceeding, ***p=0.0005, WT Proceeding vs Preceding, ***p=0.0086), conditioning day 2 (C; Fig. S9D; Two-way ANOVA, Proceeding vs Preceding x Genotype Interaction, p=0.1545) and test day (D; Fig. S8L; Two-way ANOVA, Proceeding vs Preceding x Genotype Interaction, p=0.2758; Proceeding vs preceding main effect *p=0.0136, Tukey’s Post-hoc test SST-loxP Proceeding vs Preceding, **p=0.0092) in WT and SST-loxP mice. (E-H) Pearson’s correlation of activity from CS+ tones to the first tone during baseline (E, Two-way ANOVA, Genotype Main Effect p=0.2047), discrimination day 1 (F, Two-way ANOVA, Genotype Main Effect p=0.2015), discrimination day 2 (G, Two-way ANOVA, Genotype Main Effect p=0.1029), and test day (H, Two-way ANOVA, Genotype Main Effect p=0.8850). (I-L) Pearson’s correlation of activity from CS− tones to the first tone during baseline (I, Two-way ANOVA, Genotype Main Effect p=0.3787), discrimination day 1 (J, Two-way ANOVA, Genotype Main Effect p=0.3842), discrimination day 2 (K, Two-way ANOVA, Genotype Main Effect p=0.1291), and test day (L, Two-way ANOVA, Genotype Main Effect p=0.1422). [file media-10.pdf]

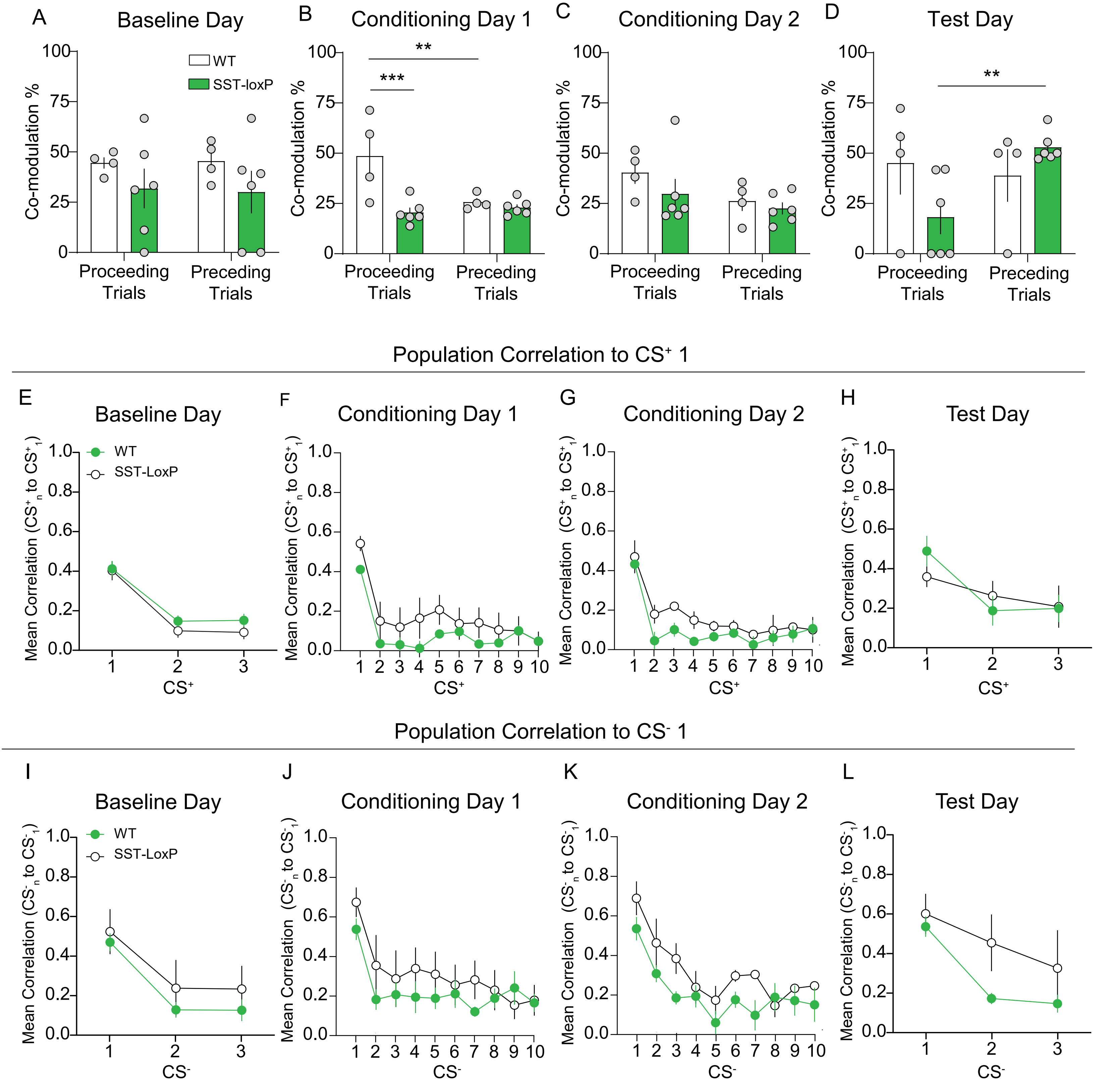
A
Baseline Day
B
Conditioning Day 1
C
Conditioning Day 2
D
Test Day
WT
SST-loxP
Co-modulation %
Proceeding Trials
Preceding Trials
***
**
Population Correlation to CS+ 1
E
Baseline Day
F
Conditioning Day 1
G
Conditioning Day 2
H
Test Day
WT
SST-LoxP
Mean Correlation (CS+n to CS+1)
CS+
Population Correlation to CS- 1
I
Baseline Day
J
Conditioning Day 1
K
Conditioning Day 2
L
Test Day
WT
SST-LoxP
Mean Correlation (CS-n to CS-1)
CS-
